# Immediate-term cognitive impairment following intravenous (IV) chemotherapy: a prospective pre-post design study

**DOI:** 10.1186/s12885-019-5349-2

**Published:** 2019-02-14

**Authors:** Omar F. Khan, Ellen Cusano, Soundouss Raissouni, Mica Pabia, Johanna Haeseker, Nicholas Bosma, Jenny J. Ko, Haocheng Li, Aalok Kumar, Michael M. Vickers, Patricia A. Tang

**Affiliations:** 1 Department of Oncology, Cumming School of Medicine, University of Calgary, Tom Baker Cancer Centre, 1331 29 St NW, Calgary, Alberta T2N 4N2 Canada; 2 Department of Medicine, University of Calgary, Calgary, Alberta Canada; 3 Margery E. Yuill Cancer Centre, Medicine Hat, Alberta Canada; 4 BC Cancer - Abbotsford, Abbotsford, British Columbia Canada; 5 Department of Mathematics and Statistics, University of Calgary, Calgary, Alberta Canada; 6 BC Cancer - Surrey, Surrey, British Columbia Canada; 7 Department of Oncology, Ottawa Regional Cancer Centre, Ottawa, Ontario Canada

**Keywords:** Drug therapy/antineoplastic combined protocols, Adverse effects, Cognitive impairment, Breast neoplasms, Colonic neoplasms

## Abstract

**Background:**

Cognitive impairment is commonly reported in patients receiving chemotherapy, but the acuity of onset is not known. This study utilized the psychomotor vigilance test (PVT) and trail-making test B (TMT-B) to assess cognitive impairment immediately post-chemotherapy.

**Methods:**

Patients aged 18–80 years receiving first-line intravenous chemotherapy for any stage of breast or colorectal cancer were eligible. Patient symptoms, peripheral neuropathy and Stanford Sleepiness Scale were assessed. A five-minute PVT and TMT-B were completed on a tablet computer pre-chemotherapy and immediately post-chemotherapy. Using a mixed linear regression model, changes in reciprocal transformed PVT reaction time (mean 1/RT) were assessed. A priori, an increase in median PVT reaction times by > 20 ms (approximating PVT changes with blood alcohol concentrations of 0.04–0.05 g%) was considered clinically relevant.

**Results:**

One hundred forty-two cancer patients (73 breast, 69 colorectal, median age 55.5 years) were tested. Post-chemotherapy, mean 1/RT values were significantly slowed compared to pre-chemotherapy baseline (*p* = 0.01). This corresponded to a median PVT reaction time slowed by an average of 12.4 ms. Changes in PVT reaction times were not correlated with age, sex, cancer type, treatment setting, or use of supportive medications. Median post-chemotherapy PVT reaction time slowed by an average of 22.5 ms in breast cancer patients and by 1.6 ms in colorectal cancer patients. Post-chemotherapy median PVT times slowed by > 20 ms in 57 patients (40.1%). Exploratory analyses found no statistically significant association between the primary outcome and self-reported anxiety, fatigue or depression. TMT-B completion speed improved significantly post-chemotherapy (*p* = 0.03), likely due to test-retest phenomenon.

**Conclusions:**

PVT reaction time slowed significantly immediately post-chemotherapy compared to a pre-chemotherapy baseline, and levels of impairment similar to effects of alcohol consumption in other studies was seen in 40% of patients. Further studies assessing functional impact of cognitive impairment on patients immediately after chemotherapy are warranted.

**Electronic supplementary material:**

The online version of this article (10.1186/s12885-019-5349-2) contains supplementary material, which is available to authorized users.

## Background

In 2017, there were an estimated 1.68 million new diagnoses of cancer in the United States [1]. In 2007, the estimated prevalence of chemotherapy treatment was nearly 650,000 individuals, with each patient having, on average, 11 annual visits during which chemotherapy was administered [2]. Cancer survivors commonly report cognitive decline after chemotherapy. This entity, often referred to as ‘chemo-brain’ or ‘chemo-fog’ [3], is described in anywhere from 17 to 78% of breast cancer patients [4]. No clear predictors of cognitive impairment after chemotherapy are currently identified [4], though older age, lower cognitive reserve and increasing chemotherapy dosage/duration are associated with cognitive decline in some studies [3, 5]. Multiple studies have shown an independent effect on cognition even after accounting for anxiety, fatigue, depression or menopause [6–8].

However, the onset and duration of cognitive decline is unclear. Some retrospective studies have shown evidence of cognitive impairment more than 20 years after chemotherapy [3], while others demonstrate improvement in cognitive impairment within months [9, 10]. Studies document onset of impairment within as little as one week of chemotherapy completion [10, 11], and Wefel et al. demonstrated the presence of impairment prior to completion of all chemotherapy cycles [12]. These studies, along with the dose-response study conducted by Collins et al., demonstrate a short-term impact on cognition with chemotherapy [5]. Some postulated mechanisms of cognitive impairment may lead to acute impairment, including increases in oxidative stress, inflammation, and decreases in hippocampal catecholamine production due to chemotherapy [13]. In addition, antineoplastic treatments are given concurrently with adjunct medications to alleviate side effects (such as diphenhydramine for breast cancer), which may independently also impact cognitive function. Cognitive impairment immediately after administration of chemotherapy therefore is important to assess on a practical level, with significant potential implications on a patient’s ability to safely perform tasks such as driving.

Overall, small study populations, heterogeneity and the presence of confounding variables limit the interpretation of data regarding chemotherapy and cognition [14], but there is reasonable neuropsychological and neuroimaging evidence that chemotherapy independently influences short-term and long-term cognitive decline. To our knowledge, however, no studies have assessed the impact on cognitive function in patients immediately (i.e. within minutes) after chemotherapy administration.

This study aimed to evaluate cognitive impairment immediately after administration of chemotherapy, utilizing surrogate cognitive tests performed on a tablet computer.

## Methods

### Study setting, design and participants

This pre-post design prospective single-site study was conducted at the Tom Baker Cancer Centre (Calgary, Canada). The study was approved by the Health Research Ethics Board of Alberta Cancer Committee. Informed consent was obtained in writing from all participants.

Patients between the ages of 18 and 80 years receiving intravenous chemotherapy for a pathologically-confirmed diagnosis of breast or colorectal cancer in the neoadjuvant, adjuvant and first-line metastatic treatment settings were eligible. Patients with a history of brain metastases, known neurological disorder (e.g. seizure disorder, prior stroke) or a history of allergic reactions to chemotherapy were excluded from the study, as were patients unable to read or understand the consent form and cognitive testing instructions. The number of prior chemotherapy cycles was recorded, as patients were not required to be chemotherapy naïve. Testing was not repeated with multiple cycles of chemotherapy for any participant.

### Symptom assessment

Participant symptoms were assessed using the Edmonton Symptom Assessment Scale [15], the Stanford Sleepiness Scale [16], and the revised Common Terminology Criteria for Adverse Events version 4.0 grading system for peripheral neuropathy [17]. Symptoms were assessed immediately prior to cognitive testing pre- and post- chemotherapy.

### Cognitive testing

The Psychomotor Vigilance Test (PVT) [18] is a test of ability to sustain attention over time, that primarily assesses orientation and attention [19, 20]. The PVT is most often used in assessment of fatigue and sleep deprivation [21, 22], and has been tested in multiple settings, including in drivers [23], pilots [24], and physicians [25, 26]. Clinically, it has been used in traumatic brain injury [27], sleep apnea [28] and to assess effects of various drugs [25, 29–31]. PVT response times significantly increase in settings of acute cognitive impairment, including alcohol intoxication and fatigue [11, 32]. While the traditional PVT is a 10-min test, shorter test durations of five minutes are also valid [19, 20, 33].

Another commonly used cognitive test is the Trail-Making Test [34], which spans multiple cognitive domains. Specifically, performance on the Trail-Making Test Part B (TMT-B) is predictive of executive function and cognitive flexibility [35]. Like the PVT, clinical and research applications of the TMT-B are widespread, including dementia [36, 37] and as part of the assessment of cognition in breast cancer patients [38]. Additionally, Day et al. demonstrated a significant correlation between breath alcohol concentrations and performance on TMT-B, indicating the usefulness of TMT-B in settings of acute cognitive impairment [39]. To minimize test-retest phenomenon with TMT-B, a previously validated mirror image of the original TMT-B form (mirrored on both the x and y axes) was utilized [40]. Participants were randomly assigned to either the original or the mirrored version of TMT-B pre-chemotherapy, and completed the alternate version post-chemotherapy. Participants were oriented to the TMT-B with a practice trail (containing eight nodes) to further minimize practice effects.

Both the PVT and TMT-B were administered via a touch-screen tablet computer, using in-house software programmed in the Java™ computer language (see Additional file 1 for details regarding test administration). Patients were tested pre-chemotherapy either immediately upon arrival to the chemotherapy unit, or in clinic the day prior to their chemotherapy infusion. Post-chemotherapy testing occurred within 15 min of completion of chemotherapy infusions at the cancer centre.

### Statistical analysis

Statistical analysis was performed using R v3.1.1 (R Foundation for Statistical Computing, Vienna, Austria). Demographics were analyzed with descriptive statistics, and linear mixed model regression analysis was utilized to assess changes in the reciprocal transformation of reaction time (mean 1/RT; decreases in mean 1/RT post-chemotherapy (compared to a pre-chemotherapy baseline) represent slowed reaction time), accounting for age, sex, cancer type, treatment setting, prior chemotherapy use, timing of testing (same-day versus prior-day), and concurrent benzodiazepine or diphenhydramine use as co-variates. Paired Wilcoxon Rank Sum tests were used to assess change in median PVT reaction time, TMT-B completion time, TMT-B errors (connections made between incorrect numbers and letters) and PVT lapses (defined as a response time > 1000 ms). A priori, an increase in median PVT reaction times by over 20 ms was considered a clinically relevant change. This change in median PVT reaction time has been shown to approximate reaction time changes with blood alcohol concentrations of 0.04 to 0.05 g% (0.5–0.8 g/L) [41]. This blood alcohol level is consistent with legal limits in jurisdictions worldwide, and was also associated with impaired attention and hazardous driving in prior studies [25, 41].

## Results

### Patient population

Between July 2014 and September 2016, 158 eligible participants consented to the study. Testing was not completed due to delays or discontinuation of chemotherapy in 10 patients. Consent was withdrawn by three patients, while one patient was excluded after consenting due to a history of stroke. Two participants experienced technical difficulties with the tablet computer, resulting in incomplete PVT and TMT data collection. Baseline characteristics of the 142 patients with complete pre-chemotherapy and post-chemotherapy testing is listed in Table 1.

**Table 1:** Demographic Data of Participants Completing Cognitive Testing Before and After Intravenous Chemotherapy (*n* = 142)

Factor	Number	Percentage
Age, years		
Average	54.7	
Median	55.5	
Range	30–80	
Gender		
Male	42	29.6
Female	100	70.4
Cancer Type		
Breast Cancer	73	51.4
Colorectal Cancer	69	48.6
Treatment Setting		
Neoadjuvant	19	13.4
Adjuvant	80	56.3
Metastatic/Palliative	43	30.3
Prior Chemotherapy Exposure		
Chemotherapy Naïve	17	12.0
Prior Chemotherapy Exposure	125	88.0
Pre-Chemotherapy Testing Timing		
Same day as chemotherapy	106	74.6
Day prior to chemotherapy	36	25.4
Antineoplastic Regimen Used		
*Breast Cancer Regimens*		
5-Fluorouracil/Epirubicin/Cyclophosphamide (FEC)	21	14.8
Docetaxel (D)	21	14.8
Docetaxel/Cyclophosphamide (DC)	14	9.9
Paclitaxel (P)	11	7.7
Docetaxel/Carboplatin (DCARB)	4	2.8
Doxorubicin/Cyclophosphamide (AC)	2	1.4
*Colorectal Cancer Regimens*		
5-Fluorouracil/Leucovorin/Oxaliplatin (FOLFOX)	45	31.7
5-Fluorouracil/Leucovorin/Irinotecan (FOLFIRI)	11	7.7
Capecitabine/Oxaliplatin (CAPOX)	9	6.3
5-Fluorouracil/Leucovorin (FUFA)	2	1.4
Irinotecan	2	1.4
Additional Antineoplastic Medications Used		
Trastuzumab	16	11.3
Bevacizumab	12	8.5
Pertuzumab	2	1.4
Panitumumab	2	1.4
Supportive Medications Used		
Steroids	142	100.0
Benzodiazepines^a^	0	0.0
Diphenhydramine	54	38.0

^a^61 patients (43.0%) had benzodiazepines available for use within their chemotherapy order sets, but medication administration records documented no use of benzodiazepines; 5 patients (3.5%) had separate benzodiazepine prescriptions, use of which would not be documented in the electronic health record

### Psychomotor vigilance test

**Fig. 1:**
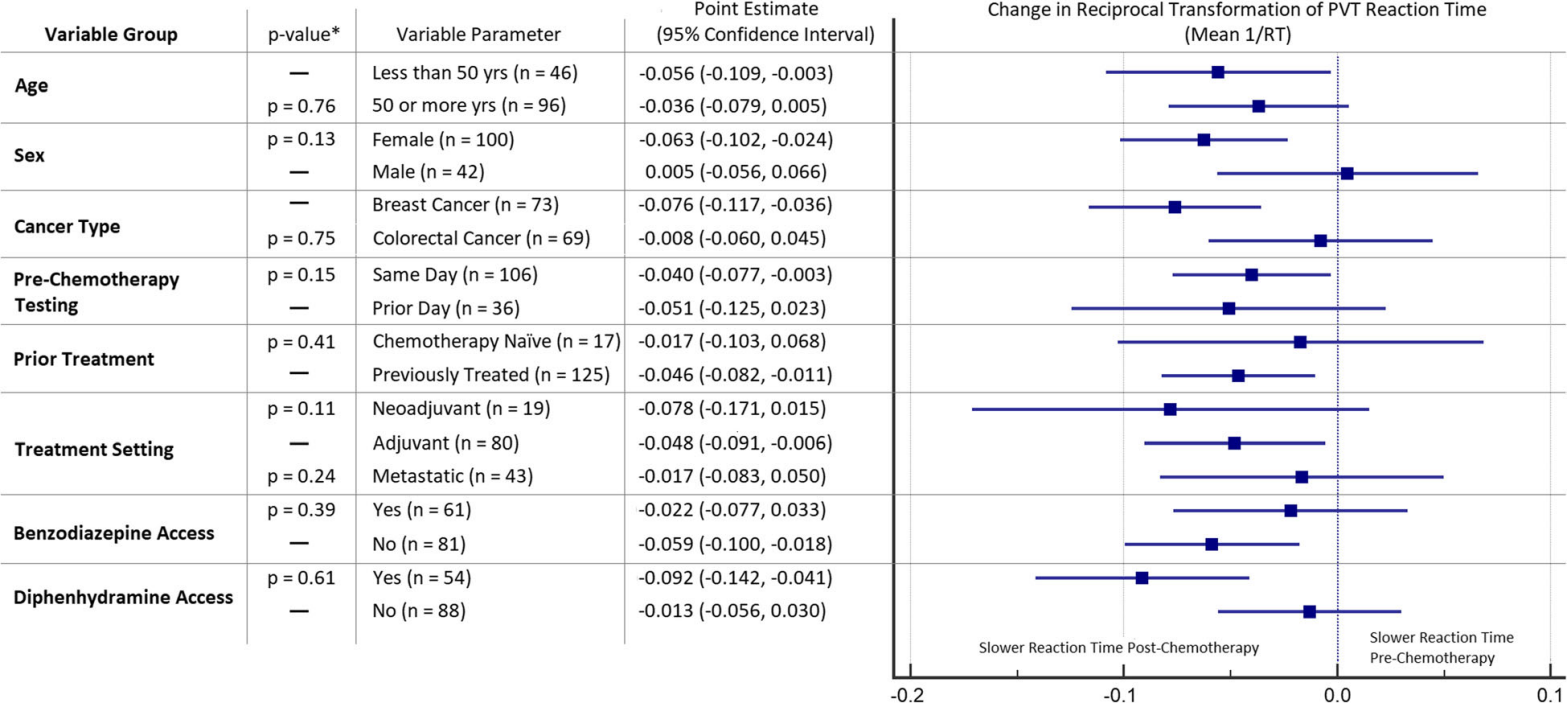
Forest plot of changes in reciprocal transformed PVT reaction time (mean 1/RT) immediately after chemotherapy administration. Calculated *p*-values for each covariate are based on a linear mixed regression model

**Fig. 2:**
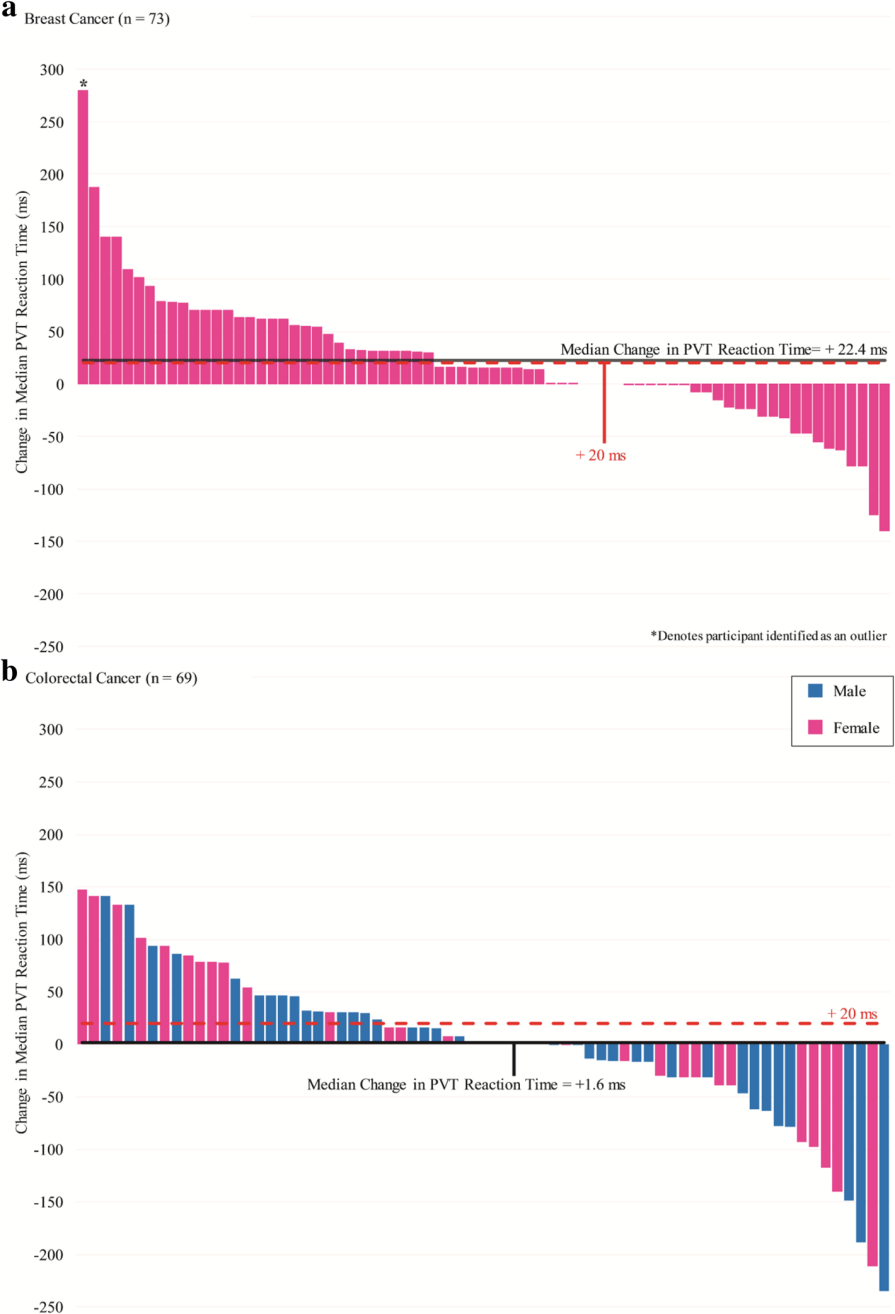
Waterfall plot of changes in median PVT reaction time in (**a**) breast cancer and (**b**) colorectal cancer patients. *Legend for* Fig. 2*:* Positive changes represent *slowed* reaction times post-chemotherapy compared to a pre-chemotherapy baseline. The red dashed line represents a slowing of 20 ms, similar to PVT reaction time changes seen with blood alcohol concentrations between 0.5–0.8 g/L in other studies [25, 41]

Compared to a pre-chemotherapy baseline, the mean reciprocal transformed PVT reaction time was significantly slowed post-chemotherapy (*p* = 0.01). Post-chemotherapy performance remained worse after adjusting for age, sex, cancer type, prior chemotherapy use, timing of pre-chemotherapy testing, or patient access to benzodiazepines or diphenhydramine (Fig. 1). Median PVT reaction time post-chemotherapy slowed by an average of 12.4 ms (*p* = 0.01). For breast cancer patients, median PVT reaction time slowed by an average of 22.4 ms, while colorectal cancer patients experienced slowing of their median PVT reaction time by an average of 1.6 ms. Figure 2 demonstrates a waterfall plot of change in median PVT reaction time, with slower times post-chemotherapy represented by positive bars, and faster times post-chemotherapy represented by negative bars. There were no differences seen in the number of lapses during the PVT post-chemotherapy compared to pre-chemotherapy (*p* = 0.845). Upon further analysis, a single outlier was identified (noted with an asterisk in Fig. 2).

A total of 58 patients (40.1%) had a clinically significant slowing in their median PVT reaction time post-chemotherapy of more than 20 ms. Of these patients, 32 were breast cancer patients (representing 43.8% of the breast cancer patients tested), while 26 were colorectal cancer patients (representing 37.7% of the colorectal cancer patients tested).

Exploratory analyses (see Additional file 2) did not reveal any statistically significant correlation between the change in mean reciprocal transformed or median PVT reaction time and participant reported fatigue, depression, anxiety or sleep scores. There was also no correlation between chemotherapy drug class or potential use of benzodiazepines and change in median PVT reaction time. Changes in mean reciprocal transformed and median PVT reaction times remained statistically significant after a sensitivity analysis removing patients with home benzodiazepine prescriptions (*n* = 5, 3.5%). There was a trend towards a correlation between diphenhydramine use and slowing of median PVT reaction times (*p* = 0.06), though the primary outcome (mean 1/RT) did not significantly interact with diphenhydramine use (*p* = 0.61).

### Trail-making test part B

Post-chemotherapy, completion of TMT-B was faster by an average of 6.7 s compared to a pre-chemotherapy baseline (*p* = 0.03). No significant main effects for cancer type, sex, prior chemotherapy use, timing of pre-chemotherapy testing, or patient access to benzodiazepines or diphenhydramine were seen (see Additional file 3). There were no differences seen in the number of errors made during TMT-B post-chemotherapy compared to pre-chemotherapy (*p* = 0.39).

Exploratory analyses (see Additional file 2) did not reveal any correlation between change in TMT-B completion time and self-reported participant fatigue, depression, anxiety or sleep scores.

## Discussion

This is, to our knowledge, the first prospective study assessing cognitive impairment in individuals immediately after receiving an intravenous chemotherapy infusion. Overall, this study demonstrated a statistically significant slowing of PVT reaction time post-chemotherapy compared to baseline, including after adjusting the model for covariates. However, a trend suggesting breast cancer patients had a more significant slowing in their median PVT reaction times as compared to colon cancer patients was observed, but did not reach statistical significance. Additionally, a sizeable proportion of participants in this study had median PVT reaction times slowed by greater than 20 ms, similar to changes seen with blood alcohol concentrations between 0.5–0.8 g/L. Disconcertingly, the change in median PVT reaction time did not correlate with participant self-assessment of fatigue, sleepiness or other cognitive symptoms. This implies that despite clinically significant changes in surrogate cognitive tests, participants were unaware of potential limitations in their cognitive function. While this finding is consistent with literature demonstrating lack of awareness of acute cognitive impairment due to fatigue or substance use (e.g. alcohol) [25, 42, 43], it may have important lifestyle and safety implications for cancer patients undergoing chemotherapy, perhaps most importantly with regards to safety surrounding driving after chemotherapy. While many comprehensive guidelines – such as the Canadian Medical Association’s Driver’s Guide [44] and a guidance released by the UK Driver and Vehicle Licensing Agency [45] – review a plethora of medical conditions and drugs, guidelines surrounding a patient’s ability to drive after chemotherapy are not included in these documents. Regarding activities such as driving, it is crucial to note that surrogate tests such as the PVT are only part of a comprehensive assessment and would not on their own be considered a valid predictor of vehicle collisions [46]. Additionally, while a 20 ms slowing in median PVT reaction time was defined a priori as clinically relevant based on studies assessing alcohol consumption [41], our study did not have a control group not receiving chemotherapy, limiting the interpretability of this finding. Therefore, evidence of slowing PVT reaction time alone would not justify withdrawing a patient’s ability to drive.

Multiple mechanisms are postulated to account for cognitive impairment after chemotherapy, including increases in oxidative stress and inflammation, as well as decreases in brain vascularization, neurogenesis and catecholamine production due to chemotherapy [13]. Patient factors such as increasing age and lower cognitive reserve are also associated with longer-term cognitive impairment after chemotherapy in some studies [3]. While our study results did not appear to be impacted by various patient factors and self-reported symptoms, a non-statistically significant trend towards association of median PVT reaction time with the use of diphenhydramine was noted. These results remained consistent after repeating testing with outliers removed. However, this finding was not confirmed in the primary mixed linear regression model analysis of mean 1/RT. Median PVT reaction time, while more intuitive to interpret, is considered inferior to the reciprocal transformed PVT reaction time for assessment of fatigue [19]. Therefore, while this exploratory finding is of interest, further research is required to clarify whether the mechanism of impairment is due to chemotherapy itself or to supporting medications such as diphenhydramine. It is important to note, however, that it is not ethically possible to withdraw diphenhydramine from a taxane-containing regimen due to hypersensitivity reaction risk [47], and from a practical viewpoint, the finding of cognitive impairment immediately after chemotherapy administration may have important functional implications for patients irrespective of the mechanism by which they occur.

In contrast to the PVT results, participants were significantly faster at completing TMT-B post-chemotherapy compared to their pre-chemotherapy baseline. Despite the use of standardized practice runs and utilization of the mirror-image TMT-B forms, this finding is most likely a result of significant test-retest phenomenon. This was confirmed anecdotally by multiple patients, some of whom noted they spent their chemotherapy infusion time practicing the sequence of connections (1 ➔ A ➔ 2 ➔ B, etc.), in an attempt to improve upon their pre-chemotherapy time.

This study was a single-centre study with limited sample size, decreasing the power to assess for patient factors impacting changes in median PVT reaction time. As the study was designed to generate hypotheses regarding acute-onset cognitive impairment after chemotherapy, a broad range of patients (in terms of treatment setting and number of chemotherapy cycles) were recruited. All patients were tested post-chemotherapy within 15 min of completion of their infusion. Additionally, this study demonstrates the feasibility of utilizing tablet computers or other handheld devices to assess patient-reported outcomes as well as objective cognitive impairment. However, each patient was only tested once, and thus the cumulative effect of multiple cycles of chemotherapy were not assessed. As cognitive testing was performed on a tablet computer, PVT reaction times were a function of the participant’s actual reaction time, in addition to the time required to perform a mechanical action (tapping the tablet screen with a stylus pen). As different patients held the tablet computer differently, this mechanical time varied for each participant, but was minimized by asking each participant to use the tablet in a similar fashion pre-chemotherapy and post-chemotherapy. Additional variation in computer processing time was minimized by using a single dedicated tablet computer for all testing. Some degree of sample bias was introduced by excluding patients unable to understand instructions for TMT-B (this was more likely to exclude non-Caucasian patients and recent immigrants); this bias was minimized by attempting to use simple symbols, colors, and clear fonts during cognitive testing.

## Conclusions

This study revealed that median PVT reaction time was significantly slower immediately after a chemotherapy infusion compared to a pre-chemotherapy baseline, and that impairment potentially correlating to the effects of alcohol was seen in 40.1% of patients. The results of this study may have important functional consequences for patients, particularly with regards to activities such as driving. Future studies should evaluate longitudinal changes in cognitive function spanning from initiation to completion of an adjuvant regimen. This would help determine whether an acute impact on cognitive function occurs consistently with each cycle, or if it changes over time. Additional research is needed to determine the duration of the immediate effect post-chemotherapy – if the effect lasts for minutes only, the implications are quite different than if the effect lasts for several hours or longer. Finally, further exploration of the functional impact of this acute change in cognitive function after administration of chemotherapy is warranted, including studies involving more comprehensive driving assessments and/or simulators.

## Additional files

Additional file 1: Detailed description of testing procedures (DOCX 17 kb)

Additional file 2: **Table S1.** Exploratory analyses of data (DOCX 17 kb)

Additional file 3: **Figure S1.** Forest plot of changes in trail-making-test-B (TMT-B) completion time immediately after chemotherapy administration, compared to a pre-chemotherapy baseline. (TIF 340 kb)
