# Supplementary material for: Immediate-term cognitive impairment following intravenous (IV) chemotherapy: a prospective pre-post design study
Source: BMC Cancer. 2019 Feb 14;19:150. doi: 10.1186/s12885-019-5349-2 (PMC6375158; doi:10.1186/s12885-019-5349-2)
Supplement: Supplementary file 1 — Detailed description of testing procedures (DOCX 17 kb) [file 12885_2019_5349_MOESM1_ESM.docx]

**Additional File 1 – Testing Procedure Details**

*Testing Environment, Equipment and Procedures*

The first component of testing involved a subjective assessment of participant symptoms and sleepiness. Sleepiness was assessed using the Stanford Sleepiness Scale. Additionally, a series of questions from the Edmonton Symptom Assessment Scale provided information regarding other symptoms, including nausea, anxiety, pain, depression and overall well-being. Peripheral neuropathy was scored using the revised NCIC-CTC criteria. This subjective assessment occurred during the pre-chemotherapy testing as well as post-chemotherapy sessions.

A researcher then provided the participant with a touch-screen tablet computer and stylus pen. A program – written and developed in-house using the Java™ computer programming language – was developed to administer and score the Psychomotor Vigilance Task (PVT) and the Trail-Making Test Part B (TMT-B). Each task occurred in sequence.

The first task for participants was the PVT. In this task, participants were asked to monitor a large yellow rectangle displayed on the tablet computer screen. The computer randomly generated a stimulus (a prominent red circle with text stating ‘Tap the Screen!’) overlying the rectangle, at time intervals set to a minimum of 2 000 ms and a maximum of 10 000 ms, as described in other studies using the PVT. Participants were asked to touch the tablet screen with their stylus pen as soon as they saw the stimulus. Once the participant pressed the screen, a “response recorded” message appeared for 1 000 ms and the participant’s reaction time (in ms) was recorded. The timer and yellow rectangle background then reset for the next trial. If the screen was not touched within 1000 ms of the stimulus appearing, a chime sounded to re-alert the participant, and the trial was recorded as a “lapse” (while traditionally PVT lapses are defined as responses taking > 500 ms, due to the nature of the touchscreen tablet – which requires a larger motor movement than a push button attached to a desktop computer – a larger lapse time definition was utilized in this study. Finally, to discourage participants from randomly tapping the screen, if the screen was tapped without a stimulus present, an error message displayed on-screen. The PVT continued for a total duration of five minutes, averaging approximately 35-40 trials per session. Additionally, participants were given a three-trial practice session, the recommended learning curve for the PVT established in previous research.

The second task for participants was Part B of the Trail-Making Test. This task asked participants to sequentially trace a path using the stylus pen through a series of 25 squares, 12 of which were labelled with letters (A through L) and 13 of which were labelled with numbers (1 through 13). Participants were required to start at the square labelled “1”, and then alternate between ascending letters and numbers (e.g. 1-A-2-B-3…) until they reached the square labelled “13”. If participants made an error (e.g. drawing a connection directly from the square labelled “1” to the one labelled “2”), the connection they drew automatically erased (though any correct connections made prior to this remained in place). The total time (in seconds) required to complete the task was recorded, along with the number of errors made. The task had a maximum time limit of 300 seconds (five minutes), after which it terminated (scoring the participant as a time of 300 seconds). To orient participants to the task, a practice trail (with only 8 squares) was provided. To alleviate practice effects, a mirror image of the original TMT-B form (reflected in both the x and y axes) was utilized. This alternate version of the TMT-B is also validated and has a retest reliability of 0.86 compared to the original TMT-B form. Participants were randomly assigned to take either the original or the mirror-image version in the pre-chemotherapy setting, and then took the alternate version post-chemotherapy.

Pre-chemotherapy testing occurred in either the outpatient clinic waiting room area or the medical day treatment area of the Tom Baker Cancer Centre (depending on the participant’s location at time of recruitment to the study). Post-chemotherapy testing occurred within 15 minutes of completion of the chemotherapy infusion at the cancer centre.
