# Supplementary material for: Immediate-term cognitive impairment following intravenous (IV) chemotherapy: a prospective pre-post design study
Source: BMC Cancer. 2019 Feb 14;19:150. doi: 10.1186/s12885-019-5349-2 (PMC6375158; doi:10.1186/s12885-019-5349-2)
Supplement: Supplementary file 2 — Table S1. Exploratory analyses of data (DOCX 17 kb) [file 12885_2019_5349_MOESM2_ESM.docx]

**Additional File 3. Supplementary Table**

**Supplementary Table 1**. Exploratory analysis for self-reported symptoms for changes in post-chemotherapy reciprocal transformed psychomotor vigilance task (PVT) reaction time (n = 142).

| **Symptom** | **Factor** | **Number of Participants^1^** | **Change in Mean 1/RT (1/s)^2^** | **95% Confidence Interval** | **p-value** | **Change in TMT Completion Time (s)^3^** | **95% Confidence Interval** | **p-value** |
| --- | --- | --- | --- | --- | --- | --- | --- | --- |
| **Self-Reported Pre-Chemotherapy Symptoms^4^** | | | | | |  |  |  |
| *Fatigue* |  | *137* |  |  | *0.92* |  |  | *0.27* |
|  | None | 42 | -0.032 | (-0.089, 0.025) |  | 9.28 | (3.35, 15.22) |  |
|  | Mild | 55 | -0.054 | (-0.103, -0.005) |  | 4.33 | (-5.55, 14.21) |  |
|  | Moderate/Severe | 42 | -0.032 | (-0.107, 0.043) |  | 4.90 | (-7.10, 16.91) |  |
| *Depression* |  | *138* |  |  | *0.98* |  |  | *0.86* |
|  | None | 96 | -0.046 | (-0.087, -0.006) |  | 5.98 | (-1.46, 13.41) |  |
|  | Mild | 32 | -0.035 | (-0.093, 0.023) |  | 6.47 | (-1.14, 14.08) |  |
|  | Moderate/Severe | 10 | -0.021 | (-0.220, 0.177) |  | 3.91 | (-9.71, 17.52) |  |
| *Anxiety* |  | *137* |  |  | *0.79* |  |  | *0.52* |
|  | None | 76 | -0.033 | (-0.076, 0.011) |  | 5.73 | (-3.14, 14.60) |  |
|  | Mild | 50 | -0.039 | (-0.089, 0.011) |  | 9.04 | (3.28, 14.80) |  |
|  | Moderate/Severe | 11 | -0.036 | (-0.231, 0.160) |  | 0.45 | (-14.07, 14.98) |  |
| *Sleepiness^5^* |  | *138* |  |  | *0.60* |  |  | *0.21* |
|  | None | 4 | 0.054 | (-0.192, 0.301) |  | -21.75 | (-142.94, 99.44) |  |
|  | Mild | 102 | -0.047 | (-0.082, -0.012) |  | 10.00 | (4.31, 15.69) |  |
|  | Moderate/Severe | 32 | -0.039 | (-0.131, 0.053) |  | -3.97 | (-15.86, 7.92) |  |
| **Self-Reported Change in Symptoms (Post- versus Pre-Chemotherapy)^6^** | | | | | |  |  |  |
| *Fatigue* |  | *131* |  |  | *0.54* |  |  | *0.25* |
|  | Improved | 46 | -0.022 | (-0.084, 0.040) |  | -0.46 | (-11.29, 10.38) |  |
|  | Unchanged | 45 | -0.075 | (-0.134, -0.017) |  | 12.78 | (4.90, 20.66) |  |
|  | Worsened | 40 | -0.033 | (-0.098, 0.030) |  | 7.37 | (-3.90, 18.63) |  |
| *Depression* |  | *131* |  |  | *0.92* |  |  | *0.42* |
|  | Improved | 25 | -0.027 | (-0.120, 0.065) |  | 6.20 | (-2.42, 14.82) |  |
|  | Unchanged | 100 | -0.046 | (-0.085, -0.007) |  | 6.89 | (-0.27, 14.05) |  |
|  | Worsened | 6 | -0.077 | (-0.283, 0.129) |  | 0.83 | (-24.65, 26.32) |  |
| *Anxiety* |  | *130* |  |  | *0.54* |  |  | *0.21* |
|  | Improved | 40 | -0.037 | (-0.110, 0.035) |  | 8.49 | (0.70, 16.27) |  |
|  | Unchanged | 82 | -0.032 | (-0.070, 0.005) |  | 6.61 | (-1.76, 14.98) |  |
|  | Worsened | 8 | -0.139 | (-0.376, 0.099) |  | -5.37 | (-19.13, 8.38) |  |
| *Sleepiness* |  | *135* |  |  | *0.78* |  |  | *0.85* |
|  | Improved | 13 | -0.026 | (-0.192, 0.141) |  | 6.69 | (-2.85, 16.23) |  |
|  | Unchanged | 75 | -0.047 | (-0.086, -0.009) |  | 9.71 | (1.70, 17.71) |  |
|  | Worsened | 47 | -0.030 | (-0.095, 0.035) |  | 0.73 | (-8.62, 10.08) |  |

^1^Some data was missing from patient questionnaires.
^2^A negative change in mean reciprocal transformed PVT reaction time represents *slowing* of reaction time post-chemotherapy compared to a pre-chemotherapy baseline.
^3^Positive values represent faster TMT-B completion post-chemotherapy compared to pre-chemotherapy.
^4^Participants grouped based on Edmonton Symptom Assessment Scale scores into “none” (0), “mild” (1-3), or “moderate/severe” (4-9). Moderate and severe groups were combined due to small sample sizes.
^5^Participant sleepiness grouped based on Stanford Sleepiness Scale as “none” (0), “mild” (1-2), or “moderate/severe” (3-7).
^6^Changes in symptoms based on post-chemotherapy worsening, improvement or no change compared to pre-chemotherapy testing.
