# Supplementary figures and images for: Immediate-term cognitive impairment following intravenous (IV) chemotherapy: a prospective pre-post design study
Source: BMC Cancer. 2019 Feb 14;19:150. doi: 10.1186/s12885-019-5349-2 (PMC6375158; doi:10.1186/s12885-019-5349-2)

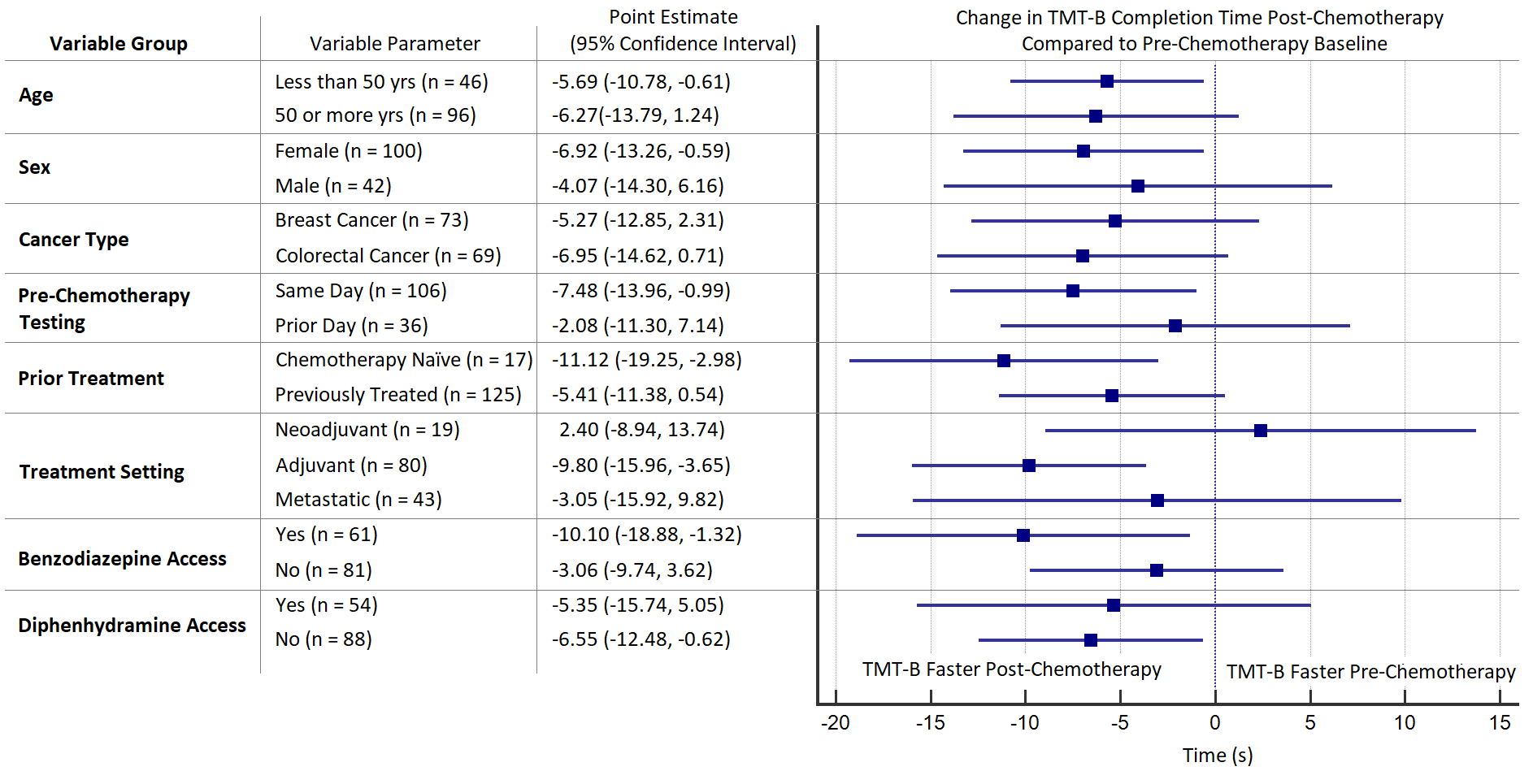

Supplement: Supplementary file 3 — Figure S1. Forest plot of changes in trail-making-test-B (TMT-B) completion time immediately after chemotherapy administration, compared to a pre-chemotherapy baseline. (TIF 340 kb) [file 12885_2019_5349_MOESM3_ESM.tif]
